# Order-Induced Selectivity Increase of Cu_60_Pd_40_ in the Semi-Hydrogenation of Acetylene

**DOI:** 10.3390/ma6072958

**Published:** 2013-07-16

**Authors:** Matthias Friedrich, Sebastián Alarcón Villaseca, László Szentmiklósi, Detre Teschner, Marc Armbrüster

**Affiliations:** 1 Max-Planck-Institut für Chemische Physik fester Stoffe, Nöthnitzer Straße 40, 01187 Dresden, Germany; E-Mails: friedrich@fhi-berlin.mpg.de (M.F.); sebasdux@gmail.com (S.A.V.); 2 Budapest, Hungary; E-Mail: szentmiklosi.laszlo@energia.mta.hu; 3 Fritz-Haber-Institute of the Max-Planck-Society, Faradayweg 4-6, 14195 Berlin, Germany; E-Mail: teschner@fhi-berlin.mpg.de

**Keywords:** Cu_60_Pd_40_, intermetallic compound, alloy, semi-hydrogenation, phase transition

## Abstract

The two structural modifications of Cu_60_Pd_40_ were synthesized as bulk powders and tested as unsupported model catalysts in the semi-hydrogenation of acetylene. The partly ordered low-temperature modification (CsCl type of structure) showed an outstanding ethylene selectivity of >90% over 20 h on stream while the disordered high-temperature modification (Cu type of structure) was 20% less selective, indicating an influence of the degree of order in the crystal structure on the catalytic properties. The results are supported by XRD and *in situ* XPS experiments. The latter suggest the existence of partly isolated Pd sites on the surface. *In situ* PGAA investigations proved the absence of metal hydride formation during reaction. Quantum chemical calculations of the electronic structure of both modifications using the CPA-FPLO framework revealed significant differences in their respective density of states, thus still leaving open the question of whether the degree of structural order or/and the electronic hybridization is the decisive factor for the observed difference in selectivity.

## 1. Introduction

The semi-hydrogenation of acetylene to ethylene is not only an important cleaning step in the industrial polymerization of ethylene but also serves as a model reaction for the exploration and development of catalysts because of the small molecules involved [1,2]. Several concepts have been applied in the past years to overcome the low selectivity of Pd catalysts towards ethylene [3,4,5].The active site isolation concept—avoiding different reaction paths, thus increasing the selectivity by hindering different adsorption geometries on the catalyst surfaces—has proven to have the biggest influence on selectivity when applied using unsupported Ga–Pd intermetallic compounds [6,7]. The unsupported intermetallic compounds GaPd, GaPd_2_ and Ga_7_Pd_3_ were shown to outperform established catalytic systems regarding ethylene selectivity and long-term stability. The improved catalytic properties in the semi-hydrogenation of acetylene on these model catalysts were attributed to structural isolation of the active sites as well as strong modification of the electronic structure compared to elemental Pd. Nevertheless, comparing compounds within a binary system is associated with the variation of several parameters that have to be considered like crystal structure, electronic structure and elemental ratio, thus complicating the assignment of the parameter having the major influence on the catalytic properties. A refined approach to possibly overcome this issue is to compare the catalytic properties of different structural modifications of a binary compound, meaning the equality in composition, and thus, alike charge transfer. However, how strongly the different crystal structures are influencing the electronic structures has to be explored case by case. Binary Pd-containing compounds with group 3–15 metals, forming intermetallic compounds showing different structural modifications, are not abundant. To our knowledge, only Al, Cu, Fe, Mn, Ti, Tl and V are known to fall within this category [8]. Recently, Zn has been disproved to form a high-temperature modification with Pd at equiatomic composition [9]. Regarding stability against oxidation or decomposition, Cu is the most noble metal among these metals, making it an appropriate candidate to study the catalytic properties of intermetallic compounds with different structural modifications. The majority of the solid part in the Cu–Pd phase diagram constitutes of complete miscibility of Cu and Pd, forming a substitutional alloy—a solid solution with mixed site occupation (Cu-type of structure, *Fm3m*) [10]. At low temperatures the solid solution decomposes into several intermetallic compounds. At a Cu:Pd atomic ratio of 3:1 the structural changes by the ordering of the *fcc* solid solution are relatively small, since the formed Cu_3_Au type of structure is just an ordering variant of *fcc*. In contrast, around the 1:1 composition, the structural changes are large. Below 600 °C and at Cu-contents of 53–65 at.-%, an partially ordered intermetallic compound with the CsCl-type of structure (space group *Pm3m*) is thermodynamically stable, realizing copper-rich compositions by mixed occupation of the Pd site by Cu and Pd [11].

Cu–Pd materials have been investigated as potential catalysts in several reactions. The two above-mentioned modifications were tested as unsupported catalysts in the decomposition of formic acid, showing significantly lower activation energy for the partly ordered intermetallic compound compared to the disordered solid solution [12]. Both modifications have also been tested in the transformation of para-H_2_ to ortho-H_2_, again revealing a lower activation energy for the transformation on the partly ordered intermetallic compound [13]. Disordered, unsupported Cu–Pd alloys with different compositions have been tested as catalysts in the hydrogenation of ethylene, showing similar catalytic properties compared to elemental Pd as long as the Cu concentration is not exceeding 53% [14]. Alumina-supported Cu–Pd catalysts have been investigated in the hydrogenation of acetylene, showing an increase in C_2_H_4_ selectivity compared to Pd/Al_2_O_3_ [15,16,17]. Because of the low metal loading, no structural information on the formed bimetallic component could be provided. Furthermore, supported Cu–Pd catalysts or Cu–Pd nanoparticles have been tested in dimethyl ether reforming [18], the water gas shift reaction [19], electrochemical oxidation of methanol [20], nitrate hydrogenation [21,22], hydration of acrylonitrile [23,24], hexa-1,5-diene hydrogenation [25] and the reduction of nitrates [26].

In this contribution we report on the preparation of the disordered solid solution and the partly ordered intermetallic compound at the composition Cu_60_Pd_40_ as unsupported powders and their catalytic properties in the semi-hydrogenation of acetylene. A detailed powder X-ray diffraction (XRD) study of the materials is supported by *in situ* X-ray photoelectron spectroscopy (XPS) and *in situ* prompt gamma activation analysis (PGAA) that focus on surface states and bulk hydrogen chemistry, respectively. Electronic structure calculations of both modifications conducted within the charge self-consistent linear-combination-of-atomic-orbitals coherent-potential-approximation (LCAO-CPA) are presented in this study. The influence of the density of states of the different modifications on the catalytic properties is discussed.

## 2. Results and Discussion

**Figure 1:**
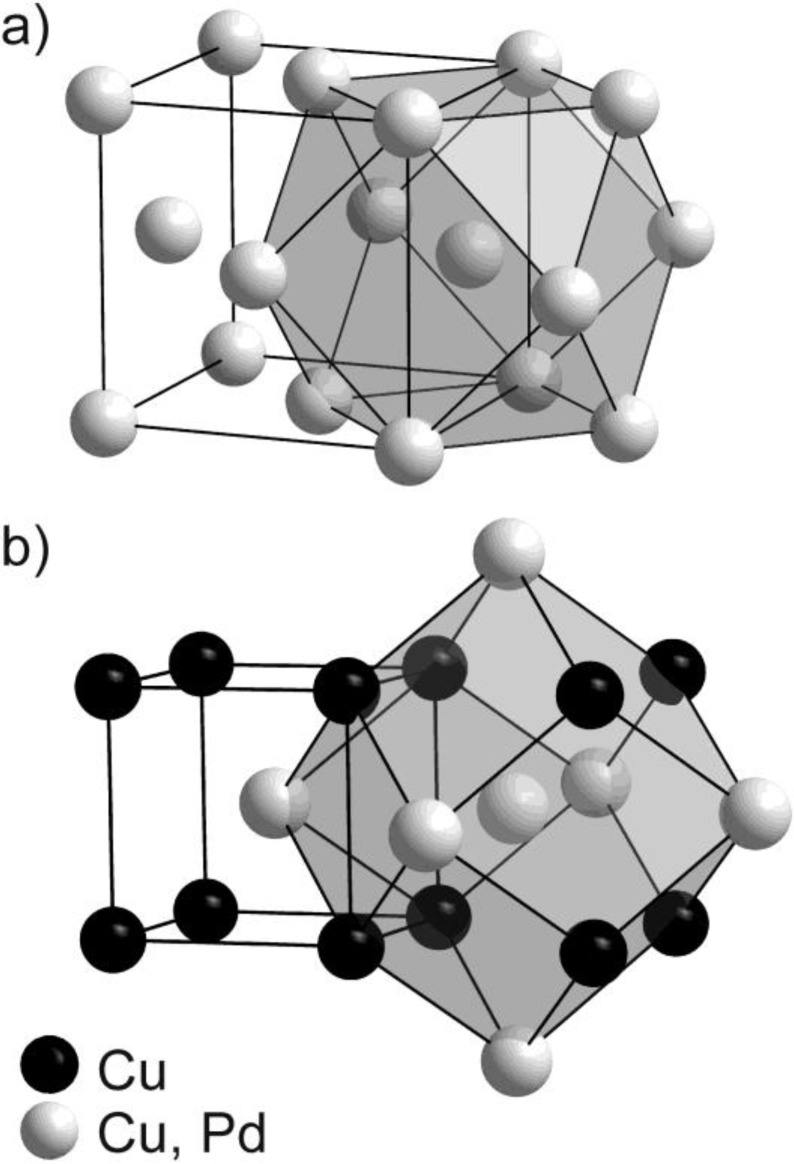
Crystal structure representation highlighting the different coordination polyhedra of (**a**) disordered Cu_60_Pd_40_ (Cu-type, *Fm3m*, *a* = 3.739(1) Å); and (**b**) partly ordered Cu_60_Pd_40_ (CsCl-Type, *Pm3m*, *a* = 2.9624(1) Å).

It is interesting to compare the two structural modifications of Cu_60_Pd_40_ concerning their active-site isolation—with a focus on palladium as presumably active species. In the high-temperature modification, *i.e.*, the *alloy*, representing an *fcc* solid solution, the atoms are randomly distributed on the crystallographic site 4*a*. This leads to a situation where each palladium atom is surrounded by 12 nearest neighbors in a cuboctahedral arrangement, out of which statistically 4.8 are palladium atoms while the others are copper atoms (Figure 1). On the other hand, in the partly ordered CsCl-type modification of Cu_60_Pd_40_, palladium atoms are sharing the central position in the unit cell, which is exclusively surrounded by eight copper atoms as first nearest neighbors, forming a cube. The six next nearest positions represent the centers of the adjacent cells which are occupied by 80% Pd and 20% Cu. This shifts the statistical 4.8 Pd–Pd contacts to the second nearest neighbors and elongates the closest possible Pd–Pd distance from 2.6436 Å in the *fcc* solid solution to 2.9624 Å in the partly ordered low-temperature modification—an increase of 12%. The better isolation is further reflected considering the Cu–Pd distance of only 2.5655Å in the ordered structure, screening the Pd atoms efficiently from each other. With respect to a possible site isolation of Pd atoms the crystal structure of partly ordered Cu_60_Pd_40_ (in the following referred to as “ordered modification”) reveals higher potential to be a selective catalyst compared to disordered Cu_60_Pd_40_.

### 2.1. Synthesis and Phase Transformation

**Figure 2:**
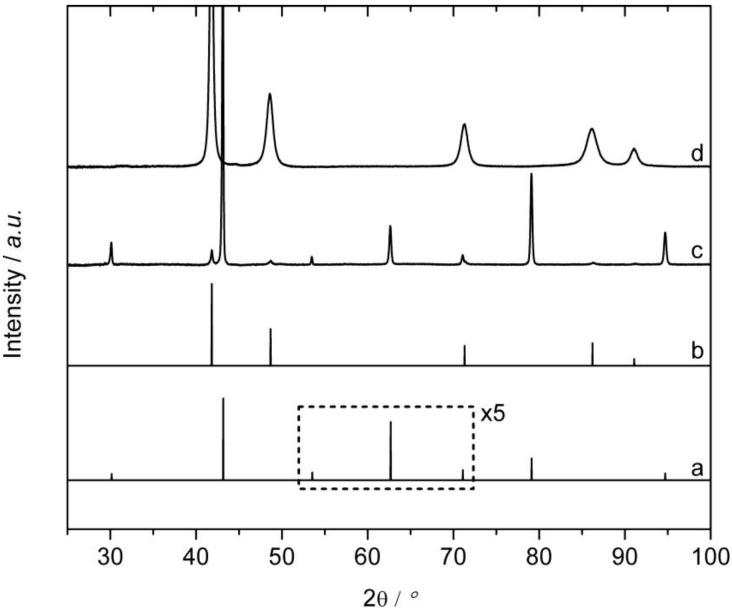
X-ray diffraction patterns: (**a**) calculated for ordered Cu_60_Pd_40_ (*a* = 2.9624 Å); (**b**) calculated for disordered Cu_60_Pd_40_ (*a* = 3.739 Å); (**c**) measurement of ordered Cu_60_Pd_40_ (annealed at 200 °C); and (**d**) measurement of disordered Cu_60_Pd_40_ (annealed at 800 °C).

The high temperature modification of Cu_60_Pd_40_—which will be referred to as the “disordered modification” from now on—was obtained as single phase sample according to the described synthesis (see Experimental Section) as shown by XRD in Figure 2d, which only shows the reflections of the disordered modification (Cu-type, *Fm3m*, *a* = 3.739(1) Å). Using the Scherrer-equation, a crystallite size of 30 nm was determined from the full-width at half maximum (FWHM) of the reflections. Subsequently, the atomic Cu:Pd ratio was determined by ICP-OES as 61.6(3):38.2(3), including Fe impurities from the stainless steel file of around 0.14 at.-%. The route to synthesize the low temperature modification of Cu_60_Pd_40_ also included filing of the compact sample after annealing at 200 °C. Surprisingly, the XRD pattern of the freshly filed powder only contained the disordered modification (not shown). Apparently, the mechanical impact due to filing caused an energetic impact sufficiently high to immediately transform the ordered into the disordered modification. This effect, often referred to as stress-induced phase transformation, is a widely known phenomenon for solid compounds forming structural modifications [27,28]. The back-transformation could be achieved by thermal treatment, which has already been proven for nanocrystalline powders of Cu_60_Pd_40_ [29]. Hence, the powder sample was enclosed into an evacuated quartz glass ampoule and annealed at 200 °C for one week. The XRD pattern (Figure 2c) confirmed that a transformation to the ordered modification (CsCl-type, *Pm3m*, *a* = 2.9624(1) Å) occurred due to annealing of the powder at 200 °C, resulting in a crystallite size of 70 nm for the ordered modification. Nevertheless, the transformation was not complete; a fraction of about 8 wt.-% of disordered Cu_60_Pd_40_ remained. Furthermore, the reflections became much sharper after annealing, which is most probably due to the healing of structural defects. No sintering of the particles was observed. The composition of the sample showing the ordered structure was determined by ICP-OES as Cu_61.5(2)_Pd_38.4(2)_, including Fe impurities of around 0.14 at.-%, thus being identical to the composition of the sample with the disordered structure.

**Figure 3:**
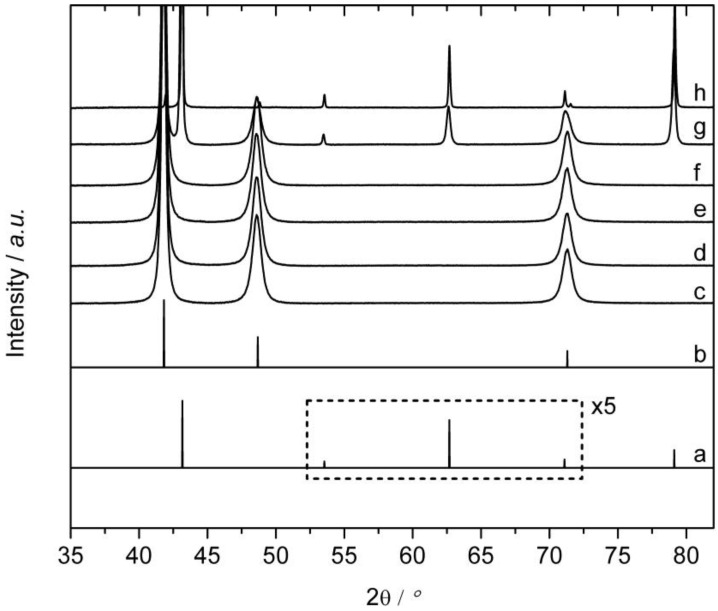
Calculated X-ray diffraction patterns of (**a**) ordered Cu_60_Pd_40_ (*a* = 2.9624 Å); (**b**) disordered Cu_60_Pd_40_ (*a* = 3.739 Å); (**c**–**h**) show the disorder-order transition for a disordered Cu_60_Pd_40_ sample annealed at 200 °C for (**c**) 2 h; (**d**) 4 h; (**e**) 8 h; (**f**) 16 h; (**g**) 32 h; and (**h**) 114 h.

Since the catalytic experiments are carried out at 200 °C, it is of high importance to determine the speed with which the sample possessing the disordered structure (annealed at 800 °C) transforms into the ordered structure representing the stable modification at 200 °C. Therefore, annealing studies of powder of disordered Cu_60_Pd_40_ were conducted in evacuated quartz glass ampoules at 200 °C for different times. Figure 3 shows that no sign of transformation was observed until 16 h of annealing. The crystallite size increased slowly during annealing from 55 nm (2 h) to 65 nm after 4–16 h. After 32 h, the sample has partially transformed into the ordered modification. As can be seen from the sharper reflections of the ordered structure (crystallite size 85 nm), the transformation apparently proceeded at the expense of small crystallites possessing the disordered structure to form larger crystallites possessing the ordered structure. After 114 h of annealing, the majority of the sample consists of ordered Cu_60_Pd_40_ (crystallite size 95 nm), only a minority is composed of disordered Cu_60_Pd_40_. As result, a catalytic test of 20 h should be appropriate to investigate the catalytic properties of the disordered modification due to the very slow transition of the disordered to the ordered modification.

### 2.2. Electronic Structure Calculations

**Figure 4:**
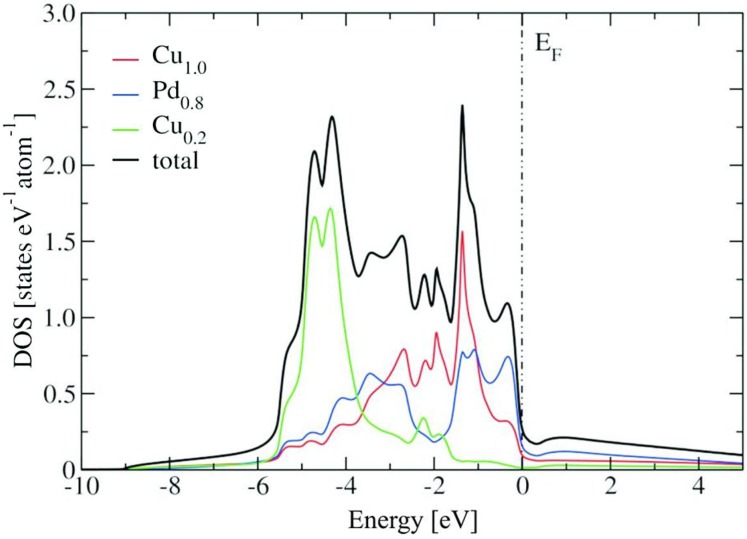
Total density of states (DOS) of the ordered Cu_60_Pd_40_ structure (Fermi level (E_F_) set to zero). Partial atomic contributions are also depicted.

Figure 4 shows the bulk density of states (DOS) of the ordered Cu_60_Pd_40_modificationwith respect to the Fermi level (E_F_), as well as its partial atomic contributions. The quantum mechanical calculations are based on experimental data for both modifications. For the partly ordered modification the 1*a* site is fully occupied by copper (named Cu_1.0_), while the 1*b* site is occupied by 20% copper (Cu_0.2_) and 80% palladium (Pd_0.8_) to account for the composition Cu_60_Pd_40_. The partial atomic contribution of Cu_1.0_ and Cu_0.2_ (Pd_0.8_) is mainly dominated by their 3d-states (4d-states), resulting in the d-block for the total bulk DOS between −5.4 and −0.2 eV. The partial contributions from the Cu_1.0_-3d, Cu_0.2_-3d and Pd_0.8_-4d states present differences: while Cu_1.0_-3dcontributes in the total bulk DOS mostly with the peaks at −1.4, −2.0 and −2.8 eV, Cu_0.2_-3d contributes mostly to the peak structures observed at −4.4 and −4.8 eV. The closest peak of the total DOS to E_F_ (at −0.3 eV) is mainly a Pd_0.8_-4d contribution. Particularly, common peaks are observed between the Pd_0.8_-4d and the Cu_1.0_-3d, between Pd_0.8_-4d and the Cu_0.2_-3d, as well as between Cu_0.2_-3d and Cu_1.0_-3d. As for many other intermetallic compounds, in the total bulk DOS a pseudo-gap (valley-like shape) is observed near E_F_ (0.97 states/eV/cell at E_F_).

**Figure 5:**
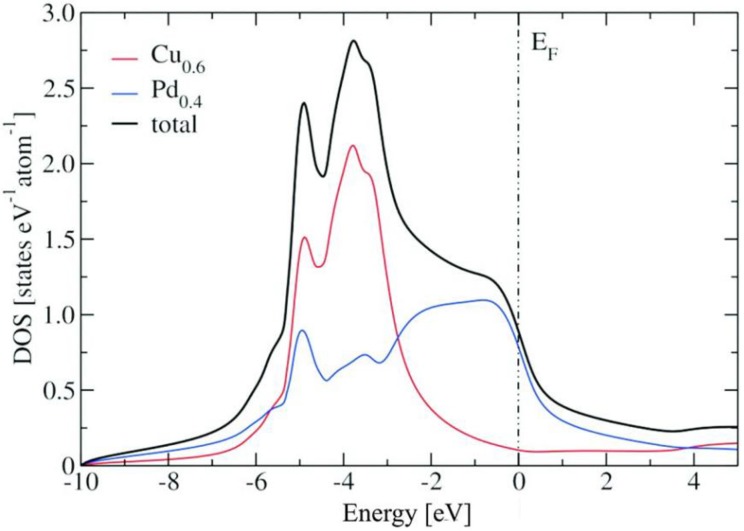
Total density of states (DOS) of the disordered Cu_60_Pd_40_ structure (Fermi level (E_F_) set to zero). Partial atomic contributions are depicted.

Figure 5 shows the density of states (DOS) of the disordered Cu_60_Pd_40_ modification with respect to the Fermi level (E_F_), as well as its partial atomic contributions. Here again, the partial atomic contribution of Cu_0.6_ (Pd_0.4_) is mainly dominated by its 3d-states (4d-states), with a band width for the total bulk DOS between −5.8 and 0.3 eV. The total bulk DOS of the disordered Cu_60_Pd_40_ modification presents a structure with two main peaks (at −3.8 and −4.9 eV) and two shoulders at −0.7 and −5.7 eV. The peak at −3.8 eV corresponds mostly to a Cu_0.6_-3d contribution, while the shoulder structure at −0.7 eV is a pure Pd_0.8_-4d contribution. Common structures of the partial DOS from Cu_0.6_-3d and Pd_0.8_-4d are observed at −4.9 (peak in total DOS) and −5.7 eV (small shoulder in total DOS), accounting for the mixing (hybridization) of these two sets of electronic states. The total DOS of the disordered Cu_60_Pd_40_ structure presents a reduction near E_F_ (1.77 states/eV/cell at E_F_), but to a minor degree and has no pseudo-gap as in the ordered Cu_60_Pd_40_ structure.

**Figure 6:**
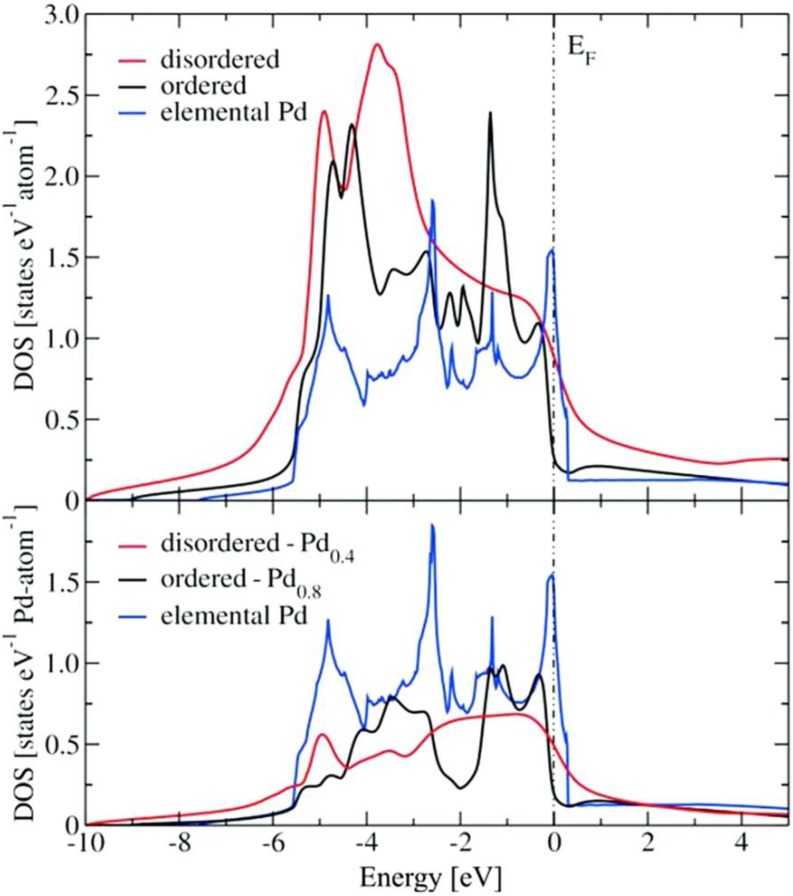
Total density of states (DOS) of the ordered and disordered Cu_60_Pd_40_ modifications (top panel, Fermi level (E_F_) set to zero). Pd contributions from both modifications are depicted in the bottom panel. The DOS of elemental Pd is shown for comparison.

Figure 6 shows a comparison between the DOS of both modifications, including their corresponding Pd contributions. Although in both ordered and disordered Cu_60_Pd_40_ modifications there are indications of hybridization, as well as a reduction of the DOS near E_F_, these findings are less pronounced in the disordered structure. Moreover, the electronic structure changes drastically between the ordered and disordered structure when going from −3 to 0.5 eV (see Figure 6). Particularly, the peaks at −0.3 and −1.4 eV observed in the ordered structure are not present in the disordered structure, suggesting important differences in their corresponding catalytic properties. This is further corroborated by comparing the Pd d-states in the vicinity of the Fermi energy. While in the ordered modification nearly all d-states are below E_F_, because of the higher Cu–Pd interaction, these states are peaking at E_F_ for elemental palladium. The disordered alloy represents an intermediate situation, *i.e.*,the d-states are falling off with a smaller slope than for elemental Pd, but still with a much higher DOS at E_F_ compared to the ordered modification. Significant differences are observed between the electronic structures of both modifications and elemental palladium (Figure 6). The degree of structuring of the ordered modification is more pronounced, which is caused by the lower dispersion of the bands in the band structure. The resulting hybridization indicates a higher degree of covalent bonding interactions in the ordered modification. The strong alteration of the electronic structure is further displayed by the stronger reduction of the DOS near E_F_ of the ordered modification.

Differences between the ordered and disordered structure are also observed when going from −3 to 0.5 eV (see Figure 6). The peaks at −0.3 and −1.4 eV, present in the ordered modification, are absent in the disordered modification. Comparison of the Pd d-states in the vicinity of the Fermi energy reveals that in the ordered modification nearly all d-states are below E_F_ due to the higher Cu–Pd interactions, while these states are peaking at E_F_ for elemental palladium. The disordered alloy represents an intermediate situation, *i.e.*, the d-states are falling off with a smaller slope than for elemental Pd, but still with a much higher DOS at E_F_ compared to the ordered modification. In general, since the chemical reactivity results from the interaction between the electronic states of the adsorbate and the electronic structure of the catalyst, the comparison suggest a different behaviour in the corresponding catalytic properties of the ordered and the disordered modification. In particular, the specificity of the positions of the electronic states that the ordered modification presents is an interesting feature that can play an important role in terms of selectivity.

### 2.3. Catalysis

**Figure 7:**
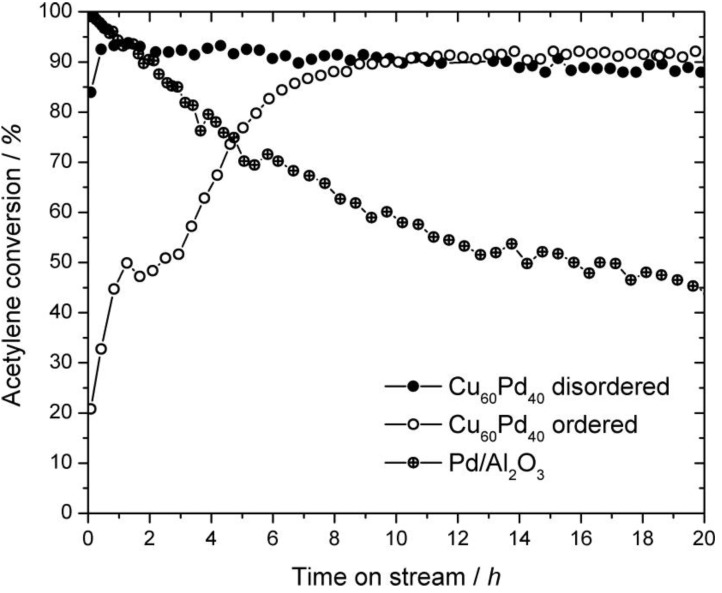
Acetylene conversion of ordered and disordered Cu_60_Pd_40_ (5 mg each) and 5% Pd/Al_2_O_3_ (0.5 mg) in the semi-hydrogenation of acetylene at 200 °C (C_2_H_2_:H_2_:C_2_H_4_ = 1:10:100). The catalysts were tested without any pretreatments. The experimental error lies within the radii of the depicted symbols.

Samples comprising the ordered or the disordered modification were tested as unsupported powders in the semi-hydrogenation of acetylene. The powders were prepared under argon and transferred to the reactor setup without contact to air. No pretreatments were conducted. Both samples show high acetylene conversion even after 20 h on stream (Figure 7). Disordered Cu_60_Pd_40_ is highly active from the beginning and suffers only a small deactivation over 20 h, going from 94% to 87%. Ordered Cu_60_Pd_40_ shows a distinct activation behavior during the first 10 h on stream. Since the ordered modification was obtained after annealing of the filed powder at 200 °C in evacuated quartz glass ampoules (see 2.1), a thin oxide layer from adhesive water might have formed. Its removal with time on stream is a possible explanation for the low initial conversion. Besides, slight reorganization of the surface can also induce an activation period. After reaching its maximum conversion, ordered Cu_60_Pd_40_ suffers no visible deactivation within 20 h on stream. In the case of disordered Cu_60_Pd_40_, sintering of crystallites leading to a reduction of the specific surface area can explain the minor deactivation. In summary, both samples show very similar specific activities (per gram catalyst), meaning that the different crystallite sizes do not influence the activity. The latter is to be expected, since the particle size in the samples is very similar due to filing of the samples.

**Figure 8:**
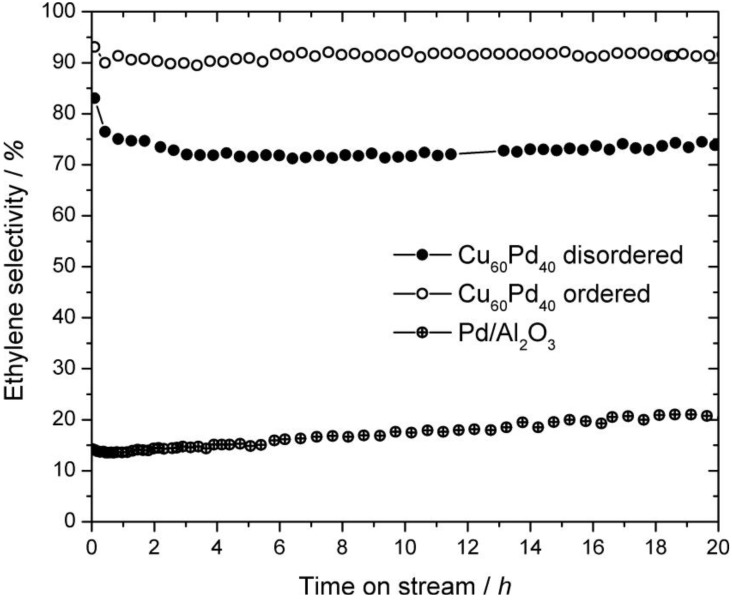
Selectivity to ethylene of ordered and disordered Cu_60_Pd_40_ (5 mg each) and 5% Pd/Al_2_O_3_ (0.5 mg) in the semi-hydrogenation of acetylene at 200 °C (C_2_H_2_:H_2_:C_2_H_4_ = 1:10:100). The catalysts were tested without any pretreatments. The experimental error lies within the radii of the depicted symbols.

Comparison of the selectivity towards ethylene reveals clear differences (Figure 8). While the ethylene selectivity observed on ordered Cu_60_Pd_40_ is 90%–92% and stable during 20 h on stream, the selectivity monitored on disordered Cu_60_Pd_40_ drops from initially >75% to around 70% within 4 h on stream and slowly increases to 74% after 20 h. Other observed products were C_4_H*_x_* species with a selectivity of 3%–5% (3%–8%) on ordered Cu_60_Pd_40_ (disordered Cu_60_Pd_40_), while the remaining carbon containing product is C_2_H_6_. Hydrocarbons higher than C4 were not observed. Thus, the ordered Cu_60_Pd_40_ produced only 4%–5% ethane, the C_2_H_6_ selectivity over the disordered modification was significantly higher (20%–23%). However, even the disordered sample is far more selective than alumina supported Pd in the semi-hydrogenation of acetylene, showing only 15%–20% ethylene selectivity under identical conditions [6]. These significant differences might be explained by the differences in their crystal and electronic structures. Apparently, the surface structures that are present under *in situ* conditions depend on the respective bulk structures, thus, the active sites on ordered Cu_60_Pd_40_ are expected to be smaller compared to disordered Cu_60_Pd_40_, leading to higher ethylene selectivity. Nevertheless, possible hydride and/or carbide formation in the near-surface region can have a major influence on selectivity and need to be investigated in *ex situ* and *in situ* studies [30,31].

### 2.4. *Ex-situ* Characterization

**Figure 9:**
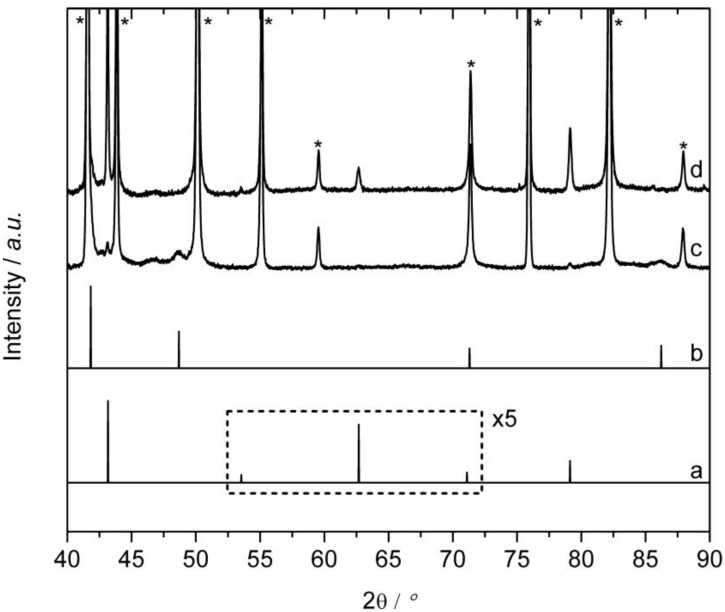
X-ray diffraction pattern of (**a**) theoretical ordered Cu_60_Pd_40_ (*a* = 2.9624 Å); (**b**) theoretical disordered Cu_60_Pd_40_ (*a* = 3.739 Å); (**c**) disordered Cu_60_Pd_40_ after catalysis; and (**d**) ordered Cu_60_Pd_40_ after catalysis. Stars indicate reflections of boron nitride used as diluent.

To investigate potential changes in the bulk material tested in the semi-hydrogenation of acetylene, the samples were removed from the reactor after 20 h on stream, brought to air and examined by XRD (Figure 9). The major component found in the XRD patterns of both catalyst samples is the inert diluent boron nitride (indicated by stars in the XRD patterns), which could hardly be separated from the Cu_60_Pd_40_ powders. After 20 h on stream, the ordered modification still exists without any transformation to the disordered modification or detectable decomposition of the compound (Figure 9d). Disordered Cu_60_Pd_40_, however, partially transformed during the 20 h on stream (Figure 9c), showing a mixture of disordered and ordered Cu_60_Pd_40_. The full width at half maximum (FWHM) of the reflections indicates that the crystallite size of disordered Cu_60_Pd_40_ is significantly smaller than the crystallite size of ordered Cu_60_Pd_40_ in this sample, thus, disordered Cu_60_Pd_40_ accounts most likely for the bigger part of the observed activity and selectivity. Furthermore, the slight increase in ethylene selectivity for the disordered modification with increasing time on stream can be interpreted as a result of the slow transformation of the disordered to the ordered modification (see 2.1).

### 2.5. *In-situ* Characterization

#### 2.5.1. PGAA

*In situ* prompt gamma activation analysis is a valuable tool to investigate the uptake of hydrogen, and thus possible hydride formation, of powder samples under reactive conditions. In the semi-hydrogenation of acetylene, elemental Pd is known to dissolve substantial amounts of hydrogen to form its *β*-hydride, which can affect the catalytic properties towards unselective hydrogenation [30]. Therefore, the absence of hydride formation is desired to obtain selective catalysts. PGAA allows for the quantification of the absorbed hydrogen content. The hydrogen uptake was investigated on a two-phase powder sample, consisting of both disordered and ordered Cu_60_Pd_40_. In pure hydrogen the atomic H/Pd ratio in the sample was determined as 0.03(3), which is negligible compared to the H/Pd ratio on elemental Pd, which is around 0.7 [30]. In a reaction mixture of H_2_:C_3_H_4_:C_3_H_6_ = 5:1:5 (used because of technical reasons), the hydrogen uptake accounted for an H/Pd ratio of 0.21(4). Nevertheless, subsequent experiments in N_2_ (H/Pd = 0.15(4)) rather point toward hydrocarbon absorption on the sample surface as a cause for the measured increase of the hydrogen signal. Thus, hydride formation seems not to take place to a larger extent in both disordered and ordered Cu_60_Pd_40_, which is in accordance to reports on hydrogen solubility in Cu–Pd thin films that do not take up any hydrogen for Pd contents ≤ 50 at.-% [32].

#### 2.5.2. XPS

**Figure 10:**
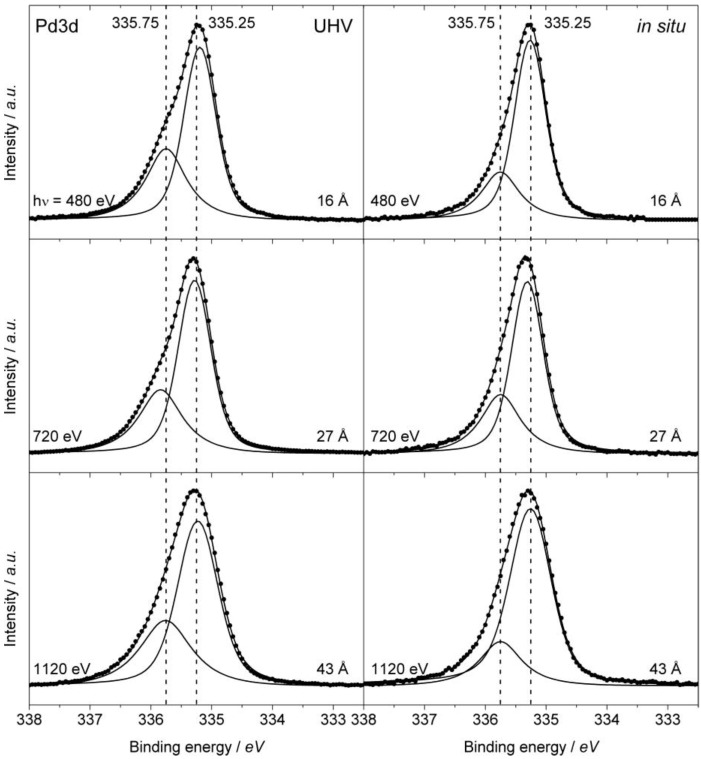
XPS Pd3d core level of ordered Cu_60_Pd_40_ in UHV (ambient temperature, 10^−8^ mbar) and under *in situ* conditions (120 °C, 0.1 mbar C_2_H_2_, 1.0 mbar H_2_). The respective photon energies and information depths are displayed.

XPS measurements on ordered Cu_60_Pd_40_ were conducted to investigate the surface of the sample regarding composition, chemical state and *in situ* stability. In general, the small amounts of Fe present in the sample from preparation (see 2.1) could not be detected in the XP spectra, corroborating the heterogeneous presence of Fe as few particles resulting from filing. Furthermore, the sample suffered severe carbon accumulation in the XPS cell in particular during *in situ* measurements, making an accurate quantification of the metal contents impossible. However, the Cu:Pd ratios were estimated to be around 1:1 at different depths under both UHV and *in situ* conditions, nevertheless, segregation phenomena could not be resolved in these experiments. The Pd3d core level spectra (Figure 10) in UHV show an asymmetric peak that is clearly comprised of two species exhibiting similar ratios at different information depths. The larger contribution originates from intermetallic palladium (Pd_IMC_), which is electronically modified by Cu atoms in the crystal structure (see Figure 1b), resulting in a shift to higher binding energies (335.25 eV) compared to elemental Pd (335 eV). Although the chemical shift is small, the difference to elemental Pd is obvious regarding the asymmetric peak shape observed on elemental Pd which was much reduced here [31]. The minor contribution at higher binding energies (335.75eV) was not reported before on ordered Cu_60_Pd_40_, which might be due to the limited resolution of the Mg *K*_α_ source [33]. The assignment to a PdC*_x_* species can be ruled out since the corresponding C1s spectra (not shown) do not exhibit the feature at 283.4 eV, typical for Pd carbides [31]. Even though the chemical nature of the Pd species at 335.25 eV, e.g. its structural relationship to elemental Pd, is not exactly known, it may be inferred that the species at 335.75 eV reflects Pd atoms that are more isolated from each other, because the larger chemical shift is induced by a more pronounced change of the electronic structure and the atomic environment, respectively. Applying *in situ* conditions induced small changes to the sample’s surface, in particular, the species at 335.75 eV decreased slightly in all investigated depths. Although quantification of the metal ratio cannot be provided in these experiments, some Pd segregation on the surface may occur in an acetylene containing feed since it is expected to interact more strongly with C_2_H_2_ than Cu [34], a phenomenon that has been observed for Cu–Pd/zeolithe samples in a CO/H_2_ atmosphere [35]. Adsorbate induced segregation was recently reviewed by Zafeiratos *et al.* [36]. This could also explain the decrease of the peak tentatively assigned as more isolated Pd species (335.75 eV). In accordance with these findings is also a recent report on the shift in binding energy due to a change in surface composition in the intermetallic compound ZnPd showing substantial zinc segregation [37]. In summary, it is difficult to draw a clear conclusion on the catalytically active surface species from the present XPS data. It can be deduced, however, that the surface of ordered Cu_60_Pd_40_ exhibits partly isolated Pd sites as would be expected from the bulk crystal structure. However, the surface slightly changes upon exposure to the reactive feed, probably by potential Pd segregation, thus somewhat reducing the amount of isolated sites at the surface.

By combining the catalytic results and XPS data, it becomes reasonable that isolated Pd atoms can aid in increasing selectivity in the semi-hydrogenation of acetylene. This hypothesis has been proven recently by investigation of single Pd atoms on Cu (111) under acetylene hydrogenation conditions by a combined high resolution STEM (scanning transmission electron microscopy) and TPD (temperature programmed desorption ) study [38]. Therein, the facile dissociation of hydrogen at Pd atom sites and its weak bonding on Cu is described, leading to a substantial change in catalytic properties compared to the pure metals.

## 3. Experimental Section

All procedures concerning synthesis were carried out in an argon-filled glove box (H_2_O and O_2_ < 0.1 ppm). To synthesize the respective Cu_60_Pd_40_ modifications elemental copper (Granules, ChemPur, 99.95%) and palladium (Powder, ChemPur, 99.95%) were weighed with an atomic ratio of Cu:Pd = 60:40 and brought to a glassy carbon crucible which was kept inside an evacuated quartz glass ampoule. The physical mixture was heated up using a high-frequency furnace (Hüttinger TIG 5/300). The temperature was raised to around 1100–1200 °C within 5–10 min according to pyrometer readings and held for another 5 min before slowly cooling down to room temperature. Massive ingots of around 2 g were obtained. To yield the disordered high-temperature and partly ordered low-temperature modifications, respectively, the samples were sealed in evacuated quartz glass ampoules and annealed in a muffle furnace at 800 °C (disordered modification) and 200 °C (ordered modification) for 2–3 weeks. Subsequently, the ampoules were quenched in water and opened in the glove box. Due to the hardness of the sample, filing was necessary to obtain powders. Impurities of the file were removed from the sample using a magnet. The synthesized powder samples as well as the samples after the catalytic tests were analyzed by X-ray diffraction (XRD) using an image plate Guinier camera (Huber, Cu-*K*_α1_, *λ* = 1.54056 Å, quartz monochromator, 3° < 2*θ* < 100°; internal standard LaB_6_, *a* = 4.15692 Å). Crystallite sizes were determined using the program Powder Cell [39]. Chemical compositions were determined by inductively coupled plasma—optical emission spectroscopy (ICP-OES) on a Vista RL (Varian). Therefore, the intermetallic samples were dissolved in aqua regia and subsequently analyzed in triplicate after matrix-matched calibration. In general, the analyzed samples showed to be lean in Pd by up to 1% compared to the initially weighted Cu and Pd amounts. This might be due to the use of fine Pd powder that is prone to stick to quartz glass surfaces during sample preparation.

X-ray photoelectron spectroscopy (XPS) using synchrotron radiation was performed at beamline ISISS-PGM at the Helmholtz Zentrum Berlin für Materialien und Energie—electron storage ring BESSY II. A detailed description of the setup can be found elsewhere [40]. 200 mg of the sample (ordered modification) were filed and pressed under argon to a pill of 8 mm in diameter and 0.5–1 mm in thickness using stainless steel pressing tools. UHV investigation of the surface of the sample was carried out at room temperature. Prior to XPS, Arion sputtering was conducted at room temperature to remove carbon layers from the surface. *In situ* experiments were carried out at 1.1 mbar and at 120 °C using a H_2_:C_2_H_2_ ratio of 10:1. Depth profiles of the samples were collected in each state of the sample (sputtered and *in situ*) by using three different photon energies for the measurement of the respective core levels (Pd 3d, Cu 3p, C 1s). For each spectrum, the Fermi edge at the corresponding photon energy was measured for energy calibration. The software Casa XPS [41] was used for qualitative and quantitative analyses of the XP spectra. To calculate Cu:Pd and metal:carbon ratios, the respective peak areas were corrected considering ring current, photon flux and tabulated cross sections [42]. Determination of the information depth was based on the calculation of the inelastic mean free path (IMFP) using the NIST Electron IMFP Database [43,44]. The information depth is three times the IMFP, thus 95% of all excited electrons originate from the respective depth [45].

*In situ* prompt gamma activation analysis (PGAA) was performed at the cold neutron beam of the Budapest Neutron Centre, Budapest, Hungary [46,47]. An Al tube reactor (inner diameter of 2 mm) loaded with 20 mg of as-prepared Cu_60_Pd_40_ powder (two-phase sample consisting of both the ordered and disordered modification) was placed into the neutron beam. The total hydrogen uptake of Cu_60_Pd_40_ was studied in pure hydrogen and in a hydrogen/propyne/propene mixture (C_3_H_4_: 0.8 mL/min; C_3_H_6_: 4 mL/min, H_2_: 4 mL/min) at around 80 °C and ambient pressure. Prompt gamma rays were collected by a Compton-suppressed high-purity germanium detector. The molar H/Pd ratio, *i.e.*, the amount of hydrogen dissolved in Cu_60_Pd_40_ and adsorbed on its surface, was determined from the characteristic peak areas corrected by the detector efficiency and the nuclear data of the elements [46,47]. Since the hydrogen spectrum contains extra contributions from gas phase hydrogen in the feed and moisture in the ‘‘viewing angle” of the detector, blank experiments were performed without Cu_60_Pd_40_ in the reactor but otherwise identical conditions to subtract the extra amount of hydrogen. Experiments were also carried out with elemental Pd for comparison [30].

Catalytic tests in the semi-hydrogenation of acetylene were conducted in a plug-flow reactor (total flow 30 mL/min) using a mixture of 0.5% C_2_H_2_ (99.6%), 5% H_2_ (99.999%) and 50% C_2_H_4_ (99.95%) in helium (99.999%) at atmospheric pressure. Prior to the catalytic runs, the Cu_60_Pd_40_ powders (5 mg) were mixed with 150 mg of catalytically inert boron nitride (AlfaAesar, 99.5% metals basis) to improve the flow characteristics in the reactor tube and to prevent the formation of hot spots. This mixture was then placed inside a reactor tube (quartz glass, inner diameter 7 mm) onto a supporting quartz glass frit. Isothermal catalytic tests were performed without any pretreatment and at 200 °C. Gas compositions were determined by a Micro GC (Varian, CP-4900) equipped with three different columns (molecular sieve, alumina, dimethyl polysiloxane) allowing for the quantification of H_2_, C_2_H_2_, C_2_H_4_, C_2_H_6_, C_4_H*_x_*, He, N_2_ and O_2_. Conversion of acetylene was calculated as:

(1) $$C(C_{2}H_{2})=\frac{c_{in}-c_{out}}{c_{in}}$$

wherein *c_in_* is the initial C_2_H_2_ concentration in the stream and *c_out_* is the amount of unconverted C_2_H_2_ in the effluent. Given the excess of ethylene, a change in ethylene concentration cannot accurately be determined. Therefore, the assumption is made that all converted acetylene is converted to ethylene in the first place (before it possibly reacts to other products). Thus, the selectivity towards ethylene is determined as

(2) $$S(C_{2}H_{4})=\frac{c_{in}-c_{out}}{(c_{in}-c_{out})+c_{C_{2}H_{6}}+2c_{C_{4}H_{x}}}$$

wherein C_C_2_H_6__ and C_C_4_H*_x_*_ are the amounts of formed ethane and C4 hydrocarbons, respectively. The selectivity towards C4 hydrocarbons is calculated following the same scheme. The carbon balance is calculated as

(3) $$CB=\;\frac{c_{out\left[C_{2}H_{2}\right]}+c_{out\left[C_{2}H_{4}\right]}\;+\;c_{out\left[C_{2}H_{6}\right]}+2c_{out\left[C_{4}H_{x}\right]}}{c_{in\left[C_{2}H_{2}\right]}+c_{in\left[C_{2}H_{4}\right]}\;+\;c_{in\left[C_{2}H_{6}\right]}}$$

wherein c*_out_* reflects the concentrations of all carbon containing molecules in the effluent and c*_in_* the initial concentrations of all carbon containing molecules. For all tested catalysts, the carbon balance accounted for ≥99%.

First-principles electronic structure calculations are performed within the local density approximation (LDA) of the density functional theory (DFT) using the version 5.00 (release 20) of the all-electron, full-potential local orbital (FPLO®) package [48]. Exchange-correlation effects are considered by employing the Perdew–Wang parametrization [49]. The semi-core and valence states, *i.e.*, (3s + 3p, 4s + 4p, 3d) for Cu and (4s + 4p, 3d, 5s + 5p, 4d) for Pd, are treated at the scalar-relativistic level. Lower-lying core states are treated employing a fully relativistic approach. A well-converged grid of 48 × 48 × 48 k-points is used to sample the Brillouin zone. The atom-centered charge densities are expanded up to l_max_ = 12. Calculations of the ordered and disordered modifications are based on the reported experimental structural parameters of the present study, while calculations of elemental Pd (Cu-type) are based on lattice parameters and atomic coordinates from [50]. To take into account the effects of the chemically disordered structure of both studied systems in the present paper, the charge self-consistent linear-combination-of-atomic-orbitals coherent-potential-approximation (LCAO-CPA), as developed and implemented by Koepernik and Eschrig [51] in the FPLO® code, is used.

## 4. Conclusions

The disordered and ordered modifications of Cu_60_Pd_40_ were synthesized and tested as unsupported model catalysts in the semi-hydrogenation of acetylene. While both compounds showed alike long-term stabilities in the catalytic reaction, the ordered modification revealed very high selectivity to ethylene (>90%), outperforming the disordered modification by around 20%. The superior catalytic properties can be attributed to the degree of ordering in the bulk crystal structure that determines the surface structure and the adsorption properties. *In situ* PGAA measurements excluded hydride formation as an influencing factor, whereas *in situ* XPS studies point towards the existence of partly isolated Pd sites at the surface that may slightly change under reactive conditions. Electronic structure calculations performed within the CPA-FPLO framework showed substantial differences in the density of states of the compounds. Particularly, the strong mixing of electronic states together with the pseudo-gap near E_F_ observed in the ordered Cu_60_Pd_40_ structure indicates a more stable and selective compound compared to the disordered structure, which is in line with the experimental findings.
